# Compensatory Health Beliefs as a Double-Edged Sword: A Dual-Path Model of Licensing and Cognitive Erosion in Multiple Health Behaviors

**DOI:** 10.3390/bs16020301

**Published:** 2026-02-20

**Authors:** Xueyi Gu, Yueqin Hu

**Affiliations:** Faculty of Psychology, Beijing Normal University, Beijing 100875, China; guxueyi@mail.bnu.edu.cn

**Keywords:** compensatory health beliefs, moral licensing, self-efficacy, physical activity, healthy eating

## Abstract

Compensatory Health Beliefs (CHBs) are cognitions that the negative effects of unhealthy behaviors can be offset by healthy ones. While their role in single behaviors is established, their mechanisms in regulating multiple health behaviors remain empirically unclear, particularly whether CHBs facilitate or inhibit actual cross-behavior compensation between physical activity (PA) and healthy eating (HE). This study tested a dual-path model proposing that CHBs are associated with immediate intention compensation via moral licensing and with long-term cross-behavior inhibition through reduced self-efficacy. A cross-sectional online survey of 366 university students assessed general CHBs, domain-specific social cognitive variables (self-efficacy, intention, planning), and self-reported PA (IPAQ-SF) and HE (calculated from reported food consumption as a dietary guideline adherence score). Data were analyzed using Structural Equation Modeling. We found that CHBs were positively associated with PA intention but negatively linked to HE intention, planning, and behavior. CHBs were also negatively related to HE self-efficacy, which was subsequently associated with lower PA planning, indicating a cross-behavior inhibition pathway. In conclusion, CHBs are linked to lower health behavior engagement through two pathways: short-term intention-based licensing across domains and self-efficacy erosion that inhibits integrated planning. This integrated model highlights the importance of addressing both CHBs and self-efficacy in health interventions.

## 1. Introduction

Physical inactivity and unhealthy diet are widely recognized as two pivotal behavioral risk factors associated with the global burden of non-communicable diseases (World Health Organization, 2010). Theoretical frameworks such as the Health Action Process Approach (HAPA) offer valuable insights into the socio-cognitive processes—including intention formation and action planning—that help bridge the “intention–behavior gap” within individual health behaviors (Schwarzer, 2008). However, these models may be limited in capturing the complexity of real-world health management, where individuals often pursue multiple health goals simultaneously, such as improving diet and increasing physical activity (Prochaska & Prochaska, 2011). In such contexts, limited cognitive resources and behavioral conflicts are likely to arise (Lippke, 2014).

Although the Compensatory Carry-over Action Model (CCAM) provides a foundational framework for understanding how CHBs may influence multiple health behaviors (Lippke, 2014), the mechanisms through which CHBs operate within networks of interacting behaviors remain poorly understood. Previous studies have reported conflicting results, with some suggesting a motivational benefit of CHBs in one domain while others point to inhibitory effects across behaviors (Radtke et al., 2014; Duan et al., 2024). These inconsistencies underscore the need to clarify the dual nature of CHBs and create a significant ambiguity regarding their fundamental role: do CHBs genuinely facilitate cross-behavior compensation between physical activity (PA) and healthy eating (HE), or do they ultimately inhibit it? This study aims to address this critical question by proposing and testing an integrative dual-pathway model.

Although the Compensatory Carry-over Action Model (CCAM) provides a foundational framework for understanding how CHBs may influence multiple health behaviors (Lippke, 2014), the precise mechanisms through which CHBs operate within networks of specific, interacting behaviors remain poorly understood. Previous studies have reported conflicting results, with some suggesting a motivational benefit of CHBs in one domain while others point to inhibitory effects across behaviors (Radtke et al., 2014; Duan et al., 2024).

To advance this literature, a productive strategy is to examine CHBs within a well-defined and functionally related dyad of behaviors. Physical activity (PA) and healthy eating (HE) represent an ideal and critical pair for this investigation. They are two of the most prevalent and modifiable lifestyle factors for chronic disease prevention, frequently co-managed in daily life and public health initiatives (Prochaska & Prochaska, 2011). Moreover, they are paradigmatic in the cultural discourse of compensation, often explicitly linked in narratives where exercise is deemed an “offset” for dietary indulgences (Cornil & Chandon, 2016; Prinsen et al., 2018). This frequent pairing in both practice and cognition makes the PA-HE dyad a prime context in which individuals’ general CHBs are likely to be applied and enacted. Consequently, the central ambiguity in the literature crystallizes into a critical empirical question within this key behavioral domain: do CHBs genuinely facilitate cross-behavior compensation between PA and HE, or do they ultimately inhibit it? This study aims to address this question by proposing and testing an integrative dual-pathway model focused on this pivotal behavior pair.

### 1.1. Theoretical Evolution: From Single to Multiple Behavior Regulation

The Health Action Process Approach (HAPA) provides a well-established framework for understanding changes in single health behaviors, framing the process within a pre-intentional motivation phase and a post-intentional volition phase (Schwarzer, 2008). During the pre-intentional stage, individuals form behavioral intentions influenced by factors such as risk perception, outcome expectancies, and self-efficacy. The subsequent volitional phase involves the translation of intentions into behavior through mechanisms such as action planning and self-regulatory control (Hagger & Luszczynska, 2014). Despite its utility, HAPA—along with other single-behavior models—exhibits limitations in accounting for competitions in cognitive resources (e.g., self-control; Hagger et al., 2010) and goal conflicts that can occur when individuals engage in multiple health behaviors concurrently (Lippke, 2014). Under such conditions, people may turn to cognitive strategies like compensatory health beliefs (CHBs) to negotiate behavioral trade-offs, a process lying beyond the explanatory scope of single-behavior frameworks.

To address this theoretical shortcoming, the Compensatory Carry-over Action Model (CCAM) was introduced as an integrative approach specifically geared toward multiple health behavior contexts (Lippke, 2014). Retaining the core “intention–planning–behavior” sequence from HAPA, CCAM incorporates two central mechanisms that help explain how behaviors may interact: (1) A carry-over mechanism, through which resources such as skills, experiential learning, or self-efficacy gained in one domain may transfer to and facilitate behavior change in another, fostering synergistic outcomes; and (2) A compensatory cognition mechanism, wherein beliefs such as CHBs emerge from perceived gaps between intentions and actions. These compensatory cognitions—often exemplified by notions such as “unhealthy eating can be compensated for by increased exercise”—may play a role in either supporting or hindering behavioral execution and may also contribute to subsequent adjustments in one’s original intentions (Lippke, 2014).

### 1.2. The Paradoxical Role of CHBs: Conflicting Evidence and Theoretical Gaps

A substantial body of empirical evidence from single-behavior studies suggests that compensatory health beliefs (CHBs) are consistently associated with negative health outcomes (Knäuper et al., 2004; M. A. Amrein et al., 2017; Fleig et al., 2015a; Fleig et al., 2015b; Glock et al., 2013; Nguyen et al., 2006). These inhibitory effects appear particularly pronounced in the domain of healthy eating, where CHBs are linked to disruptions across the entire behavior regulation sequence. For instance, CHBs may impede the formation of healthy intentions by attenuating risk perception (Ernsting et al., 2013) and may function as a self-justification strategy that weakens the translation of intentions into action (Radtke et al., 2014). These patterns have been corroborated through longitudinal research (Radtke et al., 2012) and ecological momentary assessment (M. A. Amrein et al., 2021).

In the context of physical activity, the associations involving CHBs exhibit greater complexity, suggesting a pattern of motivation–behavior decoupling. While activity-specific CHBs have been associated with reduced exercise intentions (Berli et al., 2014), their direct relationships with actual behavior often remain non-significant. Furthermore, intervention studies that successfully reduced CHBs did not yield corresponding improvements in physical activity behavior (M. Amrein & Scholz, 2016), implying that CHBs’ influence on exercise may operate through indirect pathways or intention–behavior gaps.

Notably, research indicates that the relationship between CHBs and behavioral intentions may be moderated by self-efficacy. The negative association between CHBs and intentions appears most pronounced under conditions of low self-efficacy (Storm et al., 2017), suggesting that CHBs may not merely coexist with but potentially interact with and erode essential self-regulatory resources. This provides a theoretical basis for anticipating that CHBs’ detrimental effects might extend beyond single behaviors by compromising self-efficacy that could otherwise transfer across domains.

Emerging research examining the Compensatory Carry-over Action Model (CCAM) in multiple behavior contexts has yielded both supportive and puzzling findings. For instance, Duan et al. (2024) provided empirical support for the positive carry-over mechanism by demonstrating that physical activity self-efficacy was positively associated with fruit and vegetable consumption self-efficacy.

However, they also observed a theoretically puzzling pattern: a positive association was found between diet-specific compensatory cognitions and physical activity behavior. This finding contrasts sharply with the robust evidence from single-behavior studies, which consistently links CHBs to adverse health outcomes (Radtke et al., 2014). This discrepancy may be partly attributable to a key methodological divergence; Duan et al. (2024) examined direct paths from compensatory cognitions to behavior, thereby bypassing the intention- and planning-mediated pathways through which CCAM posits such cognitions primarily operate (Lippke, 2014).

This theoretical inconsistency underscores the complexity of CHBs in multi-behavior contexts. It suggests that their effects may not be direct or uniformly negative, but could operate through more complex, undisclosed mediating pathways—potentially involving both the strategic re-regulation of intentions across domains and the gradual erosion of cross-domain self-regulatory resources.

### 1.3. Proposing a Dual-Pathway Integrative Model

To resolve the aforementioned inconsistencies, this study proposes and explores a dual-pathway model. This integrative framework posits that CHBs function as a “dual-edged sword,” operating through two concurrent mechanisms to account for the seemingly contradictory findings. Our model is built upon the robust, longitudinally validated “intention–planning–behavior” sequence that forms the core of both HAPA and CCAM (Schwarzer, 2008; Sniehotta et al., 2005; Fleig et al., 2015a). We examine how CHBs are concurrently associated with the components of these sequences across the PA and HE domains.

The model specifies: (1) a direct intention compensation pathway, whereby CHBs license short-term indulgences by providing justifications for goal-discrepant actions via moral licensing (Blanken et al., 2015; Effron & Monin, 2010); and (2) an indirect cognitive erosion pathway, whereby CHBs corrode foundational cognitive resources, specifically self-efficacy (Bandura, 1997), essential for sustaining long-term health behavior synergy within the CCAM framework (Lippke, 2014). This perspective highlights a self-perpetuating cycle in which immediate compensatory strategies may undermine the self-regulatory capacities necessary to disengage from such patterns.

#### 1.3.1. Pathway 1: The Direct Intention Compensation Pathway via Moral Licensing

Moral licensing theory suggests that initial moral acts or intentions may license subsequent indulgent behaviors by providing moral “credits” or “credentials” (Blanken et al., 2015; Effron & Monin, 2010). Within the health domain, CHBs are theorized to function as a form of moral licensing (Prinsen et al., 2018), embodying the notion that commitment to a future virtuous behavior licenses present indulgence in another domain.

The effectiveness of this strategy is thought to be influenced by the domain differentiation mechanism (Effron & Monin, 2010; Mazar & Zhong, 2010), which proposes that licensing is most effective when compensatory and compromised behaviors occur in different domains, thereby avoiding perceptions of hypocrisy. Consequently, CHBs are inherently cross-domain in nature.

This leads to a key theoretical proposition: the influence of CHBs on specific behavioral intentions may be asymmetric and role-dependent. When activated, CHBs may be associated with suppression of intention for the compromised behavior—since the immediate need for restraint is offset by the planned future compensation (Kronick & Knäuper, 2010; Miquelon et al., 2012)—and enhancement of intention for the compensatory behavior, as this intention serves as a moral credential that justifies licensing (Khan & Dhar, 2006).

To specify the probable direction of this asymmetry within the PA-HE dyad, we integrate insights regarding the behaviors’ regulatory nature and the prevailing socio-cultural context. First, the fundamental properties of HE and PA differ: HE constitutes a high-frequency, temptation-driven behavior demanding continual impulse inhibition (Hofmann et al., 2012; Cornil & Chandon, 2016), whereas PA is a lower-frequency, planned behavior relying more on proactive initiative (McEachan et al., 2011; Hofmann et al., 2012). This regulatory dichotomy positions HE as more susceptible to being the “licensed indulgence” due to its context-triggered lapses, while PA, as a discrete effortful act, is more readily framed as a potent “compensatory credential”.

Second, although the general CHBs scale measures a broad compensatory tendency across domains (e.g., stress, sleep), it does not dictate a specific PA-HE trade-off. This general belief is channeled by a dominant cultural narrative that explicitly frames exercise as an “offset” for dietary indulgences (e.g., “burning off” calories; Cornil & Chandon, 2016; Prinsen et al., 2018). Consequently, for individuals managing both diet and exercise, the readily accessible “PA-compensates-for-HE” script becomes the primary application of their CHBs. The effectiveness of this cross-domain script is further reinforced by its alignment with the domain differentiation principle (Effron & Monin, 2010).

Therefore, this integrated perspective offers a parsimonious and directional account for findings like those of Duan et al. (2024): a positive association between compensatory cognitions and PA behavior may indeed be attributable to an unmeasured increase in PA intention that functionally licenses dietary indiscretions. In testing the moral licensing pathway, we will thus specifically examine whether CHBs are associated with enhanced PA intention concurrently with suppressed HE intention.

#### 1.3.2. Pathway 2: The Indirect Cognitive Erosion Pathway via Self-Efficacy

In addition to the direct pathway involving intention compensation, CHBs are theorized to be linked to a more subtle and enduring adverse influence by potentially undermining foundational cognitive resources essential for effective self-regulation, particularly self-efficacy (Bandura, 1997). According to social cognitive theory, self-efficacy—defined as the belief in one’s capability to organize and execute courses of action required to attain desired outcomes—is most effectively developed through enactive mastery experiences (Bandura, 1997). CHBs may impede the accumulation of such critical experiences by offering a readily accessible cognitive excuse for disengagement (e.g., “I can skip this workout because I will eat healthy tomorrow”), thereby fostering avoidance of self-regulatory challenges (Radtke et al., 2012). Each instance in which an individual relies on a CHB to justify non-action may represent a missed opportunity to resist temptation or overcome barriers, which in turn could attenuate domain-specific self-efficacy. This dynamic suggests a potential cyclical process: lower self-efficacy may increase reliance on CHBs as a coping mechanism (Storm et al., 2017), while the use of CHBs may further limit opportunities for building and sustaining self-efficacy (Radtke et al., 2012).

We further propose that this erosive influence might not be confined to the behavior domain in which a CHB is initially activated. Within the Compensatory Carry-over Action Model (CCAM), self-efficacy is regarded as a transferable resource that can support behavior change across domains (Lippke, 2014). Although self-efficacy is domain-specific, Bandura (1997) noted that it can generalize across functionally related activities. Thus, reduced self-efficacy in one domain (e.g., healthy eating) could be associated with impairments in the planning and execution of a related behavior (e.g., physical activity), as a weakened sense of personal agency may extend across complementary health domains. This cross-behavioral spillover of efficacy beliefs represents a novel indirect pathway through which CHBs might disrupt integrated systems of health behavior regulation, resulting in broader inhibitory effects on behavioral synergy.

### 1.4. The Present Study and Hypotheses

**Figure 1:**
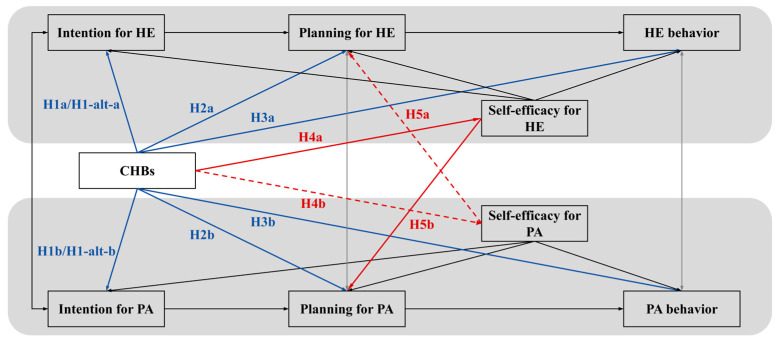
Dual-Pathway Theoretical Model of CHBs in Multiple Health Behavior Regulation. Notes. PA = physical activity; HE = healthy eating; SE = self-efficacy. This model integrates two pathways of generalized CHBs on HE/PA’s “intention–planning–behavior” sequences, plus CCAM/HAPA constructs. Black lines represent the established HAPA intention–planning–behavior sequences within each domain. Blue solid lines depict the direct intention compensation pathway (moral licensing), corresponding to H1–H3. Red lines represent the indirect cognitive erosion pathway via self-efficacy: solid red lines (CHBs → HE SE and HE SE → PA planning) illustrate H4a and H5b, which together constitute the indirect effect (H6a) of CHBs on PA planning through HE SE; dashed red lines (CHBs → PA SE and PA SE → HE planning) illustrate H4b and H5a, which together represent the hypothesized but nonsignificant indirect effect (H6b) of CHBs on HE planning through PA SE.

The present study aims to examine the associative patterns specified by the dual-pathway model (see Figure 1), using cross-sectional data to evaluate its fit and the plausibility of the proposed linkages, thereby providing a foundational framework for future longitudinal causal inquiry. Derived from the theoretical model, the hypotheses are organized around its two core pathways and their expected outcomes.

First, concerning the direct intention compensation pathway, we test the moral licensing proposition against the prevailing view from single-behavior research. The general detriment view predicts uniformly negative associations: CHBs will be negatively associated with both HE intention **(H1a)** and PA intention **(H1b)**, consistent with prior findings (Knäuper et al., 2004; Radtke et al., 2014). In contrast, the moral licensing view posits an asymmetric pattern of intention regulation. To impartially test this mechanism’s core logic—that CHBs facilitate a cross-behavior trade-off—we specify two competing directional hypotheses, acknowledging that while our analysis (Section 1.3.1) suggests the “PA-compensates-for-HE” script is more plausible, the opposite pattern is theoretically possible. Thus, **H1-alt-a** reflects an “HE-compensates-for-PA” script, predicting CHBs will be positively associated with HE intention (where HE serves as the compensatory behavior) and negatively with PA intention (where PA is the licensed behavior). **H1-alt-b** reflects the “PA-compensates-for-HE” script, predicting CHBs will be positively associated with PA intention (where PA serves as the compensatory behavior) and negatively with HE intention (where HE is the licensed behavior).

Second, irrespective of their effects on intentions, CHBs are theorized to justify goal-discrepant actions and erode self-regulatory capacity, leading to lower behavioral engagement. Therefore, we hypothesize direct negative associations with subsequent planning and behavior in both domains: CHBs will be negatively associated with HE planning **(H2a)** and PA planning **(H2b)**, as well as with HE behavior **(H3a)** and PA behavior **(H3b)**.

Third, for the indirect cognitive erosion pathway, we hypothesize that CHBs are concurrently associated with the erosion and impaired transfer of self-efficacy. Specifically, CHBs will be negatively associated with HE self-efficacy **(H4a)** and PA self-efficacy **(H4b)**. In line with the CCAM’s carry-over principle (Lippke, 2014), we expect self-efficacy in one domain to positively associate with planning in the other: PA self-efficacy with HE planning **(H5a)**, and HE self-efficacy with PA planning **(H5b)**. Building upon H4 and H5, we further hypothesize significant indirect associations, wherein the negative links between CHBs and planning are transmitted through self-efficacy in the complementary behavioral domain. Thus, the negative association between CHBs and PA planning will be mediated by HE self-efficacy **(H6a)**, and the negative association between CHBs and HE planning will be mediated by PA self-efficacy **(H6b)**.

## 2. Materials and Methods

### 2.1. Participants

A total of 373 responses were recorded. After data cleaning, 366 valid questionnaires were retained, yielding an effective response rate of 98.12%. Data cleaning was performed based on the following a priori criteria: (1) failure to correctly answer an embedded attention-check item (e.g., “To ensure you are paying attention, please select ‘Strongly Disagree’ for this statement”), and (2) an implausibly short completion time, defined as falling below two standard deviations of the mean completion time (which would indicate random responding or a lack of engagement).

The final valid sample comprised students from 54 different universities and colleges in Beijing. The sample was well-balanced in terms of gender (52.2% male, 47.8% female). Participants’ ages ranged from 18 to 27 years (*M* = 21.69, *SD* = 1.62). In terms of academic standing, the majority were undergraduates (79.2%), followed by master’s students (13.1%), and doctoral students (7.7%). Regarding personal monthly disposable income, the vast majority of participants (87.7%) reported a range between ¥1000 and ¥4000, which is representative of the typical student population in China.

### 2.2. Measures

#### 2.2.1. Health Behaviors: Physical Activity

Physical activity was measured using the short-form International Physical Activity Questionnaire (IPAQ-SF; Lee et al., 2011). This 7-item scale assesses the frequency (days) and duration (minutes) of vigorous-intensity, moderate-intensity, and walking activities over the previous seven days. A sample item includes reporting time spent in vigorous physical activities. To mitigate the established systematic overestimation bias of the IPAQ-SF, a conservative computation method was employed. Total weekly physical activity volume was expressed as metabolic equivalent (MET)-minutes/week, calculated by multiplying the MET value for each intensity (vigorous: 8.0; moderate: 4.0; walking: 3.3) by the reported daily duration, assuming participation on a single day per reported activity. Higher scores indicate greater physical activity volume.

#### 2.2.2. Health Behaviors: Healthy Eating

Healthy eating was assessed using the simplified 25-item Food Frequency Questionnaire (FFQ25; Gao et al., 2011; Gao, 2012). This scale captures intake frequency and quantity across 25 food categories relevant to chronic disease risk in Chinese populations, aided by standardized portion images. The FFQ25 has demonstrated satisfactory test–retest reliability and criterion validity in previous validation studies (Gao, 2012; Huang et al., 2022).

A healthy eating score was derived based on the Dietary Guidelines for Chinese Residents (2022). For each of the 25 food categories, individual intake was compared against the recommendations specified in the Chinese Dietary Pagoda: a score of 2 was assigned for intake within the recommended range, a score of 1 for intake exceeding the maximum recommendation, and a score of 0 for intake below the minimum recommendation. Scores across all categories were summed to create a total score ranging from 0 to 50, with higher scores indicating closer adherence to national dietary standards. This guideline-based composite score is a formative index, and as such, internal consistency estimates (e.g., Cronbach’s α) are not psychometrically appropriate for evaluating its reliability (Streiner, 2003). The validity of this score is established through its direct alignment with evidence-based national guidelines.

#### 2.2.3. Compensatory Health Beliefs

Compensatory health beliefs (CHBs) were evaluated using the validated 18-item Chinese version of the Compensatory Health Beliefs Scale (Zhang et al., 2022). The scale comprises four subscales: substance use, dietary/sleep habits, stress, and weight management. Participants rated their agreement on a 5-point Likert scale from 1 (Strongly disagree) to 5 (Strongly agree). A sample item is: “Drinking more water can compensate for the effects of eating salty foods”. Higher scores reflect stronger endorsement of compensatory health beliefs. The scale demonstrated good internal consistency in the present study (*α* = 0.871).

#### 2.2.4. Social–Cognitive Variables

Measures of self-efficacy, intention, and action planning for healthy eating (HE) and physical activity (PA) were adapted from Sniehotta et al. (2005), and Fleig et al. (2015b). All items used a 5-point Likert scale from 1 (strongly disagree) to 5 (strongly agree), with higher scores indicating stronger expression of the construct. Specifically, *Physical activity self-efficacy* was assessed with two items: “I am sure that I can do more regular physical activity, even if I have to force myself to start immediately” and “I am sure that I can do more regular physical activity, even if I feel a strong temptation not to exercise”. Correlation coefficient between the two items was 0.523. Internal consistency was *α* = 0.67. *Healthy eating self-efficacy* was assessed with two items: “I am sure that I can improve my daily nutrition, even if I have to force myself to start immediately” and “I am sure that I can improve my daily nutrition, even if I feel a strong temptation to snack and indulge”. Correlation coefficient between the two items was 0.538. Internal consistency was *α* = 0.69.

*Physical activity intention* was assessed with three items: “I intend to vigorously exercise regularly, so that I sweat and become short of breath”, “I intend to be regularly and moderately active, so that I sweat a bit in leisure time”, and “I intend to be active in daily life (walking, biking, house and garden work)”. Internal consistency was *α* = 0.41. *Healthy eating intention* was assessed with three items: “I intend to eat more fruit or vegetables”, “I intend to eat a more balanced diet”, and “I intend to adhere to the healthy Mediterranean diet”. Internal consistency was *α* = 0.65.

*Physical activity planning* was assessed with two items: “I have made a detailed plan for when, where, or how to exercise” and “I have made a detailed plan for how often and with whom to exercise”. Correlation coefficient between the two items was 0.742. Internal consistency was *α* = 0.85. *Healthy eating planning* was assessed with two items: “I have made a detailed plan for when, where, or how to eat fruit or vegetables” and “I have made a detailed plan for how to maintain an overall balanced diet”. Correlation coefficient between the two items was 0.688. Internal consistency was *α* = 0.81.

The full English versions of all scales used in this study are provided in Appendix A.

### 2.3. Procedure

This study employed a cross-sectional online survey design. Data collection was conducted over a two-week period in April 2024. Participants were recruited from universities and colleges across Beijing through a multi-channel approach, including university-affiliated online bulletin board systems, social media groups (e.g., WeChat and QQ groups dedicated to course information and student activities), and snowball sampling where students were encouraged to share the survey link with their peers.

The inclusion criteria for participants were: (1) being a full-time undergraduate or postgraduate student enrolled in a higher education institution in Beijing, and (2) being aged 18 years or older. The only exclusion criterion was a failure to provide electronic informed consent prior to participation.

All procedures were performed in accordance with the ethical standards of the university’s research committee and with the 1964 Helsinki declaration and its later amendments. Before commencing the questionnaire, all participants were presented with a detailed digital information sheet outlining the study’s purpose, procedures, potential risks and benefits, and data management policies. Electronic informed consent was obtained from all participants by requiring them to select “I have read the information above and voluntarily agree to participate in this study” before proceeding. The survey required approximately 15–20 min to complete. Upon completion, participants could receive ¥10 as a token of appreciation for their time.

### 2.4. Statistical Procedures

Data cleaning, descriptive statistics, and Pearson’s correlation analyses were conducted using IBM SPSS Statistics for Windows, version 26.0 (IBM Corp., Armonk, NY, USA). The primary analytical aim of this study was to examine the complex pattern of associations between CHBs and the social–cognitive and behavioral sequences within and across the physical activity (PA) and healthy eating (HE) domains.

To address this aim, we employed structural equation modeling (SEM) in Mplus 8.3 (Muthén & Muthén, Los Angeles, CA, USA). SEM is suited for examining the concurrent relationships among a network of variables and for evaluating how well a proposed theoretical structure fits the observed data. It is important to note that while the present cross-sectional data are used to estimate these concurrent associations, the core “intention–planning–behavior” sequences within each domain, which form the skeleton of our model, are specified based on strong longitudinal evidence from prior research (e.g., Sniehotta et al., 2005; Fleig et al., 2015a). Within this correlational design, all reported path coefficients and indirect effects are to be interpreted as statistical associations that reflect contemporaneous relationships, consistent with the proposed theoretical model. These associations provide a necessary descriptive and preliminary evidential basis for the plausibility of the model, which merits future longitudinal testing to examine temporal sequences and causality.

Consistent with this descriptive and model-evaluative aim and guided by the Compensatory Carry-over Action Model (CCAM; Lippke, 2014), a two-step modeling approach was adopted to disentangle the proposed associative patterns. First, a basic model was specified to examine the direct associations between CHBs and the social-cognitive and behavioral variables (i.e., intentions, planning, behavior) across both domains. This model primarily relates to the pattern of associations suggested by the moral licensing conceptual pathway.

Subsequently, to explore the pattern of associations corresponding to the cognitive erosion conceptual pathway, an optimized model was estimated. This model incorporated the theorized associative role of self-efficacy, aligning with the integration of CCAM and self-efficacy theory (Bandura, 1997). Specifically, paths from general CHBs to domain-specific self-efficacy and from each domain’s self-efficacy to the planning of the other behavior were added. This model specification allowed us to examine whether the observed association between CHBs and cross-behavior planning was concurrently related to self-efficacy levels, thereby exploring the pattern implied by the cognitive erosion pathway. Comparing the basic and optimized models—particularly observing changes in the magnitude of direct associations and in overall model fit—provided a means to assess how the inclusion of self-efficacy variables altered the descriptive network of associations.

Both models utilized maximum likelihood (ML) estimation to handle missing data (missing proportion <5%). Residual covariances among CHBs and behavior-specific variables (intention and planning) were permitted to account for shared variance among related constructs. Model fit was assessed using multiple indices: χ^2^/df ratio (values between 2 and 5 indicating acceptable fit; Bollen & Long, 1993), the Comparative Fit Index (CFI), the Tucker–Lewis Index (TLI > 0.95), and the Root Mean Square Error of Approximation (RMSEA < 0.05; Kline, 2005).

To robustly test the cross-behavior indirect effects posited in the dual-path model (e.g., mediation via self-efficacy), Bayesian estimation was additionally employed as a supplement to the ML analysis. This approach was chosen to enhance the robustness of the estimates for these specific paths, given the model’s complexity and the potential for small-sample bias in complex SEM with ML estimation. Bayesian methods offer more stable parameter estimates under these conditions and provide an intuitive probabilistic framework for evaluating indirect associations through 95% Credibility Intervals (Miočević et al., 2018; van de Schoot & Depaoli, 2014; Yuan & MacKinnon, 2009).

The Markov Chain Monte Carlo (MCMC) algorithm was implemented with two independent chains of 10,000 iterations each. Convergence was determined using the Potential Scale Reduction factor (PSR < 1.05; Gelman et al., 2004), and parameter estimates were summarized using medians (Miočević et al., 2018). Significance of indirect effects was evaluated based on 95% Credibility Intervals (CI) not containing zero (Yuan & MacKinnon, 2009). Model fit under Bayesian estimation was assessed using the Posterior Predictive *p*-value (PPP > 0.05) and the Deviance Information Criterion (DIC), with lower values indicating better model parsimony (Spiegelhalter et al., 2002).

## 3. Results

### 3.1. Descriptive Statistics and Correlations

Means, standard deviations, and correlations for all study variables are presented in Table 1. The mean score for the Compensatory Health Beliefs (CHBs) scale was 47.40 (*SD* = 11.35), demonstrating good internal consistency (Cronbach’s *α* = 0.87). Participants reported significantly lower physical activity intentions (*M* = 3.51, *SD* = 0.68) compared to healthy eating intentions (*M* = 4.13, *SD* = 0.65). Conservatively estimated weekly physical activity averaged 621.01 MET-min/week (*SD* = 564.03). The mean healthy eating score was 12.09 (*SD* = 1.85; maximum 50 points), a pattern consistent with structural dietary imbalances documented in the Scientific Research Report on Dietary Guidelines for Chinese Residents (Chinese Nutrition Society, 2021).

Correlational analyses revealed that CHBs were negatively correlated with healthy eating intention (*r* = −0.171, *p* < 0.01) and planning (*r* = −0.211, *p* < 0.01). Strong positive correlations were observed between domain-specific self-efficacy and corresponding intentions and planning (*rs* from 0.421 to 0.517, *p* < 0.01), supporting HAPA postulates. Physical activity planning and healthy eating planning showed high synergy (*r* = 0.764, *p* < 0.01). However, associations between planning and actual behavior were weak (physical activity planning with behavior: *r* = −0.104, *p* < 0.05; healthy eating planning with behavior: *r* = 0.127, *p* < 0.05). Significant cross-behavior correlations were found between healthy eating self-efficacy and physical activity planning (*r* = 0.433, *p* < 0.01), and between physical activity self-efficacy and healthy eating planning (*r* = 0.426, *p* < 0.01).

### 3.2. Structural Equation Modeling: Testing the Direct Pathways

**Figure 2:**
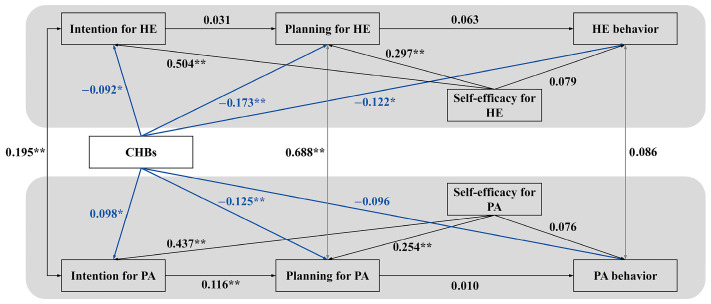
Basic Model Testing Direct Pathways of CHBs. Note. The model tests the direct associations of compensatory health beliefs (CHBs) with social–cognitive constructs and behaviors across domains, without including the self-efficacy pathways. Black lines represent the established HAPA intention–planning–behavior sequences within each domain. Blue solid lines depict the direct intention compensation pathway (moral licensing), corresponding to H1–H3. Standardized coefficients are shown. PA = physical activity; HE = healthy eating. * *p* < 0.05, ** *p* < 0.01. Key path coefficients: CHBs → HE Intention (*β* = −0.092 *), CHBs → HE Planning (*β* = −0.173 **), CHBs → HE Behavior (*β* = −0.122 *); CHBs → PA Intention (*β* = 0.098 *), CHBs → PA Planning (*β* = −0.125 *).

A sequential modeling approach was employed to rigorously dissect the proposed dual-pathway mechanisms (see Figure 2). We first specified a basic model that included only the direct paths from CHBs to the social–cognitive and behavioral variables. This initial model served as a critical baseline for two key purposes: first, to test the standalone moral licensing pathway (H1, H2, H3) by examining the direct associations between CHBs and the intention–planning–behavior sequences; second, and more importantly, to establish the total effects of CHBs before introducing the mediators (self-efficacy), thereby allowing for a clear quantification of the indirect effects in the subsequent model. The fit of this direct-effects model was suboptimal (*CFI* = 0.886, *RMSEA* = 0.128), providing initial evidence that substantive explanatory pathways were omitted. Within this basic model, several key hypotheses were supported. CHBs were negatively associated with HE intention (*β* = −0.092, *p* = 0.040), HE planning (*β* = −0.173, *p* < 0.01), and HE behavior (*β* = −0.122, *p* = 0.020), supporting H1a, H2a, H3a. Concurrently, a positive association emerged between CHBs and PA intention (*β* = 0.098, *p* = 0.037), supporting the moral licensing hypothesis (H1-alt-b). However, CHBs were also negatively associated with PA planning (*β* = −0.125, *p* = 0.010, H2b), while the direct path to PA behavior was not significant (H3b). This pattern underscores the complex, domain-specific nature of CHBs’ direct effects.

### 3.3. The Optimized Model: Integrating the Self-Efficacy Mediation Pathways

**Figure 3:**
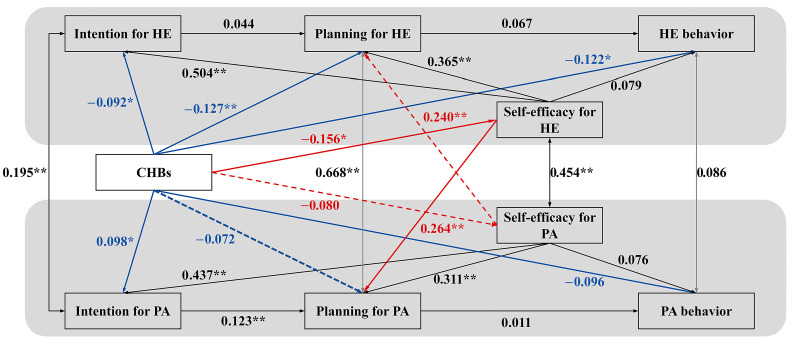
Optimized Dual-Pathway Model Integrating Self-Efficacy. Note. The model incorporates self-efficacy pathways to test the dual-process mechanisms (direct intention compensation and indirect self-efficacy erosion) underlying compensatory health beliefs (CHBs). Black lines represent the established HAPA intention–planning–behavior sequences within each domain. Blue solid lines depict the direct intention compensation pathway (moral licensing), corresponding to H1–H3. Red lines represent the indirect cognitive erosion pathway via self-efficacy: solid red lines (CHBs → HE SE and HE SE → PA planning) illustrate H4a and H5b, which together constitute the indirect effect (H6a) of CHBs on PA planning through HE SE; dashed red lines (CHBs → PA SE and PA SE → HE planning) illustrate H4b and H5a, which together represent the hypothesized but nonsignificant indirect effect (H6b) of CHBs on HE planning through PA SE. Standardized coefficients are shown. Solid lines represent significant paths (* *p* < 0.05, ** *p* < 0.01); the nonsignificant path from CHBs to physical activity (PA)-specific self-efficacy is omitted for clarity. HE = healthy eating; SE = self-efficacy. Key illustrated associations: CHBs → HE Self-Efficacy (*β* = −0.156 *); HE Self-Efficacy → PA Planning (*β* = 0.264 **); PA Self-Efficacy → HE Planning (*β* = 0.240 **). Bayesian mediation analysis confirmed the significant indirect association of CHBs with PA planning through HE self-efficacy.

To test the complete dual-pathway framework and formally evaluate the mediating role of self-efficacy (H4–H6), we incorporated the self-efficacy pathways into the optimized model (see Figure 3). The model fit improved substantially and was deemed excellent (*CFI* = 0.985, *RMSEA* = 0.054). This significant improvement in model fit confirms that the self-efficacy pathways capture essential mechanisms that the direct-effects model failed to account for.

In this integrated model, the previously observed direct effects of CHBs on HE intention, planning, and behavior remained stable and significant, further reinforcing H1. The positive link between CHBs and PA intention (H1-alt-b) also persisted. Crucially, the previously significant direct negative path from CHBs to PA planning became non-significant (*β* = −0.071, *p* = 0.101). This change indicates that the initial direct association between CHBs and PA planning was accounted for by the newly introduced self-efficacy pathways, a pattern consistent with an indirect-only mediation model.

Supporting H3a, CHBs were negatively associated with healthy eating self-efficacy (*β* = −0.156, *p* = 0.002, H4a), but not with physical activity self-efficacy (H4b). The carry-over mechanism (H5) was strongly supported, with healthy eating self-efficacy positively predicting PA planning (H5b, *β* = 0.264, *p* < 0.001) and physical activity self-efficacy positively predicting HE planning (H5a, *β* = 0.240, *p* < 0.001).

Finally, Bayesian analysis provided evidence for the core cognitive erosion pathway, revealing a significant indirect effect through healthy eating self-efficacy on the negative association between CHBs and PA planning (H6a, indirect effect = −0.004, *95% CI* [−0.008, −0.001]). The reverse pathway (H6b) was not supported.

## 4. Discussion

The present study aimed to map the complex associative patterns between general compensatory health beliefs (CHBs) and the social–cognitive sequences underlying physical activity (PA) and healthy eating (HE). By examining a dual-pathway model integrating moral licensing (Blanken et al., 2015; Effron & Monin, 2010) and self-efficacy (Bandura, 1997) theories within the CCAM framework (Lippke, 2014), our findings delineate a network of associations supporting the notion of CHBs as a dual-edged sword. The results reveal two distinct patterns: one reflecting an immediate intention trade-off across domains, and another, more pervasive pattern of associations indicative of eroded self-regulatory resources.

### 4.1. The Asymmetric Intention Pattern: Evidence for a Cross-Domain Licensing Script

The results revealed a distinct, asymmetric pattern linking general CHBs to behavioral intentions across domains: a positive concurrent association with physical activity (PA) intention alongside a negative association with healthy eating (HE) intention. This pattern directly clarifies the theoretical ambiguity presented in the introduction. It demonstrates that CHBs are not uniformly associated with lower intentions—as the general detriment view extrapolated from single-behavior research might suggest (Knäuper et al., 2004; Radtke et al., 2014)—but can co-occur with heightened intentions for a culturally sanctioned compensatory behavior. This is precisely consistent with a moral licensing script (Prinsen et al., 2018), wherein bolstering intention for one behavior (PA) serves as a cognitive credential that licenses indulgence in another (HE). The psychological plausibility of this specific “PA-compensates-for-HE” script is supported by the domain differentiation principle (Effron & Monin, 2010) and aligns with the dominant cultural narrative framing exercise as an “offset” for diet (Cornil & Chandon, 2016).

Critically, this finding provides a parsimonious account for the seemingly paradoxical positive link between compensatory cognitions and PA-related outcomes reported by Duan et al. (2024). Our results suggest that such a link may be specific to the motivational (intention) phase, reflecting a strategic enhancement of PA intention to license dietary license, rather than a direct facilitation of behavior.

However, this intention-based licensing pattern was isolated and did not translate into better self-regulatory outcomes. CHBs were concurrently associated with lower HE planning and behavior, and the positive link to PA intention did not manifest in more PA planning or behavior. Thus, the licensing script appears to function primarily as a cognitive justification for goal-discrepant actions (Kronick & Knäuper, 2010), aligning with evidence that CHBs often license indulgence rather than motivate genuine compensation (Radtke et al., 2012). The overall associative footprint of CHBs thus remained inhibitory regarding actual behavioral engagement.

### 4.2. Explaining the Divergence: Self-Regulatory Properties and the Cognitive Erosion Pathway

The observed divergence—where CHBs were linked to the entire HE sequence but primarily to the planning (rather than intention) of PA—can be understood through the interplay between the inherent properties of each behavior and the postulated cognitive erosion pathway via self-efficacy.

First, the fundamental self-regulatory demands differ. HE constitutes a high-frequency, temptation-driven behavior requiring continual impulse inhibition (Hofmann et al., 2012), making it highly vulnerable to justifications that permit immediate disengagement. Each invocation of a CHB likely represents a missed opportunity for enactive mastery—the core source of self-efficacy (Bandura, 1997). Consequently, a habitual reliance on CHBs is plausibly associated with a diminished sense of efficacy in managing one’s diet, as reflected in the significant negative association between CHBs and HE self-efficacy. In contrast, PA is a discrete, planned behavior (McEachan et al., 2011) dependent more on proactive initiative. Its self-efficacy may therefore be less immediately susceptible to erosion by the kind of momentary justifications that CHBs provide, potentially explaining the non-significant association with PA self-efficacy. This domain-specific erosion pattern aligns with the view that the detrimental correlates of CHBs are most pronounced in behaviors requiring continuous restraint (Storm et al., 2017).

Second, the self-efficacy eroded in one domain showed significant cross-behavior associations. Consistent with the carry-over mechanism posited by the CCAM (Lippke, 2014), HE self-efficacy was positively associated with PA planning, and PA self-efficacy with HE planning. This confirms self-efficacy as a transferable resource across related health domains (Bandura, 1997). More importantly, Bayesian analysis revealed that the negative association between CHBs and PA planning was accounted for by HE self-efficacy. This delineates a cross-behavior inhibitory pathway: CHBs are concurrently associated with lower self-efficacy in a particularly vulnerable domain (HE), which in turn relates to reduced planning of complementary behavior (PA). This suggests CHBs may be linked to a weakened sense of personal agency that spills over, undermining integrated health behavior management.

### 4.3. Theoretical Integration and Practical Implications

Theoretically, this study advances the integration of moral licensing and self-efficacy theories within a multiple-behavior framework. It proposes that the role of CHBs is best understood through two concurrent patterns of association: a strategic intention–regulation pattern that manifests as asymmetric cross-domain licensing, and a corrosive self-regulatory pattern wherein CHBs are linked to the erosion and impaired transfer of self-efficacy. This dual-pathway model provides a coherent framework for reconciling prior conflicting findings. It explains how CHBs can appear motivationally benign (or even facilitatory for PA intention) in simple analyses while being consistently linked to detrimental outcomes in more comprehensive models that account for planning, behavior, and the cross-domain spillover of cognitive resources.

From an intervention perspective, these insights highlight nuanced targets. First, cognitive restructuring is needed to help individuals recognize and challenge the deceptive logic of the prevalent “PA-for-HE” licensing script, reducing its potency as a justification for indulgence. Second, and critically, interventions should prioritize building domain-specific self-efficacy, particularly in vulnerable domains like healthy eating. This can be achieved through guided mastery experiences (Bandura, 1997), such as successfully navigating specific dietary temptations in a supported manner. Strengthening this core cognitive resource may serve a dual purpose: it directly facilitates behavior change within that domain and, via positive carry-over effects, may help inoculate against the cross-behavior inhibitory associations linked to CHBs. This approach is posited to be especially beneficial for individuals with low baseline self-efficacy, thereby potentially disrupting the postulated negative cycle of justification and diminished agency.

### 4.4. Limitations and Future Directions

Several limitations must be considered when interpreting these associative patterns. First and foremost, the cross-sectional design precludes causal or temporal inferences. The modeled pathways and “indirect effects” represent statistically derived associations consistent with our theoretical propositions; they provide a foundational descriptive model that must be tested longitudinally to establish temporal precedence and causal mechanisms (Maxwell & Cole, 2007; Cain et al., 2018). Building on methodological recommendations for research with limited resources (Cain et al., 2018), a practical next step would be to employ a sequential mediation design (e.g., measuring CHBs at T1, self-efficacy and intentions at T2, and planning/behavior at T3) to begin disentangling the temporal ordering implied by the dual-pathway model.

Second, the measurement of CHBs may have influenced the results. The general CHBs scale used, though reliable, contains a preponderance of items where healthy eating is portrayed as the compensatory behavior (Knäuper et al., 2004). This may have systematically primed participants, potentially reinforcing the observed “PA-licenses-HE” asymmetric pattern. Future research should utilize or develop more balanced measures that equally represent different compensatory directions to ensure a domain-neutral assessment of the construct and test the generalizability of the asymmetry.

Third, although structured instruments (FFQ25, IPAQ-SF) were used to measure concrete behaviors, all data were self-reported and thus subject to recall inaccuracies and social desirability biases. Future studies would benefit from incorporating objective measures (e.g., accelerometers, image-based dietary records) to enhance validity.

Fourth, the use of a university student sample limits generalizability. The age range, lifestyle, and health priorities of students may differ from those of other populations. Replication in more diverse community and clinical samples is essential to establish external validity.

Finally, our model is not exhaustive. Future research should explore other potential moderators (e.g., trait self-control) and mediators (e.g., goal conflict). Employing experience sampling methods could capture the dynamic, within-person processes of CHB activation and their immediate consequences, moving beyond the between-person, static associations examined here.

## Figures and Tables

**Table 1:** Means, Standard Deviations, and Correlations for All Variables (*N* = 366).

	1	2	3	4	5	6	7	8	9	10	11
1. Age	-										
2. Gender	0.027	-									
3. CHBs	0.065	0.004	-								
4. Intention for PA	−0.243 **	−0.074	0.063	-							
5. Intention for HE	0.03	0.065	−0.171 **	0.196**	-						
6. Plan for PA	−0.229 **	−0.214 **	−0.129 *	0.360 **	0.275 **	-					
7. Plan for HE	−0.174 **	−0.208 **	−0.211 **	0.266 **	0.329 **	0.764 **	-				
8. Self-efficacy for PA	−0.185 **	−0.094	−0.08	0.421 **	0.191 **	0.486 **	0.426 **	-			
9. Self-efficacy for HE	−0.057	−0.048	−0.156 **	0.146 **	0.513 **	0.433 **	0.517 **	0.460 **	-		
10. PA behavior	0.029	−0.088	0.028	−0.196 **	−0.098	−0.104 *	−0.023	−0.106 *	−0.057	-	
11. HE behavior	0.118 *	0.011	−0.148 **	−0.065	0.095	0.043	0.127 *	0.053	0.131 *	−0.065	-
*M*	21.69	1.48	47.4	3.51	4.13	3.35	3.42	3.75	3.67	621.01	12.09
*SD*	1.62	0.5	11.35	0.68	0.65	1.15	1.15	0.83	0.85	564.03	1.85

Notes: Gender: 1 = male, 0 = female; * *p* < 0.05; ** *p* < 0.01.

## Data Availability

The data that support the findings of this study are available on request from the corresponding author. The data are not publicly available due to their containing information that could compromise the privacy of research participants.

## Abbreviations

The following abbreviations are used in this manuscript:

PA	Physical Activities
HE	Healthy Eating
CHBs	Compensatory Health Beliefs
HAPA	Health Action Process Approach
CCAM	Compensatory Carry-over Action Model

## Appendix A. Measures

### Appendix A.1. Demographic Information

1. Gender: Male/Female
2. Academic Year: Freshman/Sophomore/Junior/Senior/Year 5 (for medicine, etc.)/Master’s Year 1/Master’s Year 2/Master’s Year 3
3. Average monthly expenditure during school: 600–1000 RMB/1000–2000 RMB/2000–3000 RMB/3000–4000 RMB/4000–5000 RMB/Above 5000 RMB
4. Area you lived in for most of the time before university: Urban/Rural

### Appendix A.2. Compensatory Health Beliefs Scale (CHBs)

**Table A1:** Items of the Compensatory Health Beliefs Scale (CHBs): Original English Version and Chinese Translation Used in the Study.

CHBs (English)	Chinese Version
1. The effects of regularly drinking alcohol can be made up for by eating healthy.	1. 经常喝酒的影响可以通过健康饮食来弥补
2. It is alright to drink a lot of alcohol as long as one drinks lots of water to flush it.	2. 酒喝多了也没关系，只要多喝水来冲淡酒精就可以了
3. Smoking from time to time is OK if one eats healthy.	3. 只要一个人吃得健康，时不时抽支烟也没事
4. The effects of drinking coffee can be balanced by drinking equal amounts of water.	4. 喝等量的水可以平衡喝咖啡的影响
5. The effects of drinking too much alcohol during the weekend can be made up for by not drinking during the week.	5. 周末酒喝过头的话，下周不喝酒就没事了
6. Smoking can be compensated for by exercising.	6. 吸烟的影响可以通过锻炼来弥补
7. Too little sleep during the week can be compensated for by sleeping in on the weekends.	7. 一周睡眠过少可以通过周末来补觉
8. It is OK to go to bed late if one can sleep longer the next morning.	8. 只要第二天早上晚起，头天晚上睡晚点也没事
9. It is OK to skip breakfast if one eats more during lunch or dinner.	9. 午餐或晚餐时吃点的话，早餐可以不吃
10. Eating whatever one wants in the evening is OK if one did not eat during the entire day.	10. 白天不吃东西的话，晚上想吃什么都行
—	11. 多喝水可以弥补吃得太咸的影响
12. Stress during the week can be made up for by relaxing on the weekend.	12. 一周积累的压力可在周末得到放松。
13. A stressful day can be compensated for by relaxing in front of the T.V.	13. 舒服地看电视可以补偿紧张的一天。
14. The bad effects of stress can be made up for by exercising.	14. 压力的不良影响可以通过锻炼来弥补。
15. Sleep compensates for stress.	15. 睡眠可以缓解压力。
16. Eating dessert can be made up for by skipping the main dish.	16. 不吃主食可弥补吃甜点的影响。
17. Using artificial sweeteners compensates for extra calories.	17. 使用人工甜味剂可以消减额外的卡路里。
18. Breaking a diet today may be compensated for by starting a new diet tomorrow.	18. 今天不节食，可以通过明天开始的新节食来补偿。

Note. Response Scale: Both versions used a 5-point Likert scale. The Chinese instructions were: 1 = 一点也不接近 (Not at all close), 2 = 比较不接近 (Slightly close), 3 = 一般 (Moderately close), 4 = 比较接近 (Quite close), 5 = 非常接近 (Very close). This corresponds to the degree to which each belief matches the participant’s own. Added Item: The Chinese version included one additional item (No. 11) not present in the original scale: “多喝水可以弥补吃得太咸的影响” (“Drinking more water can compensate for the effects of eating salty foods”). This addition was made for cultural relevance, addressing a common dietary context (high salt intake) in the population studied. Subsequent factor analysis confirmed its appropriate loading (>0.4) onto the intended “Eating/Sleeping Habits” factor.

### Appendix A.3. International Physical Activity Questionnaire—Short Form (IPAQ-SF)

**Table A2:** Items of the International Physical Activity Questionnaire—Short Form (IPAQ-SF).

IPAQ-SF (English)	Chinese Version
1. During the last 7 days, on how many days did you do vigorous physical activities like heavy lifting, digging, aerobics, or fast bicycling? _____ days per week No vigorous physical activities → Skip to question 3	1. 在过去的7天内，有多少天你做了像举重、有氧运动或快速骑自行车这种高强度的体育活动？ _____ 天/周 没有高强度体育活动→跳转到第3题
2. How much time did you usually spend doing vigorous physical activities on one of those days? _____ hours per day _____ minutes per day Don’t know/Not sure	2. 在以上时间中的某一天，你通常花多少时间做剧烈的体育活动？ _____ 小时/天 _____ 分钟/天 不清楚/不确定
3. During the last 7 days, on how many days did you do moderate physical activities like carrying light loads, bicycling at a regular pace, or doubles tennis? Do not include walking. _____ days per week No moderate physical activities → Skip to question 5	3. 在过去的7天里，你有多少天做了中等强度体育活动，如背负轻物，以正常的速度骑自行车，或者打网球？不包括步行。 _____ 天/周 没有中等强度体育活动→跳转到第5题
4. How much time did you usually spend doing moderate physical activities on one of those days? _____ hours per day _____ minutes per day Don’t know/Not sure	4. 在以上时间中的某一天，你通常花多少时间做适度的身体活动？ _____ 小时/天 _____ 分钟/天 不清楚/不确定
5. During the last 7 days, on how many days did you walk for at least 10 min at a time? _____ days per week No walking → Skip to question 7	5. 在过去的7天里，你有多少天一次至少走10分钟？ _____ 天/周 没有步行/散步→跳转到第7题
6. How much time did you usually spend walking on one of those days? _____ hours per day _____ minutes per day Don’t know/Not sure	6. 在以上时间中的某一天，你通常花多少时间走路？ _____ 小时/天 _____ 分钟/天 不清楚/不确定
7. During the last 7 days, how much time did you spend sitting on a week day? _____ hours per day _____ minutes per day Don’t know/Not sure	7. 在过去的7天的工作日里，你每天坐多久？ _____ 小时/天 _____ 分钟/天 不清楚/不确定

### Appendix A.4. Social–Cognitive Variables

Healthy eating behavior was assessed using the Chinese Simplified Food Frequency Questionnaire (FFQ-25), a validated tool designed to capture dietary patterns relevant to chronic disease risk in the Chinese population (Gao et al., 2011; Gao, 2012). This study utilized a digital version of the questionnaire with skip logic and integrated standardized photographic aids to facilitate accurate portion size estimation. The FFQ-25 assesses consumption frequency and typical portion size for 25 major food categories over the past week.

For each of the 25 food categories, participants answered two questions: (1) consumption frequency, and (2) typical portion size per occasion (if applicable). Frequency options ranged from “Never” to “Three or more times daily.” To improve the accuracy of self-reported portions, visual guides were provided for key food items. For example, images depicted a standard 50 g (liang) serving of staple foods (e.g., a steamed bun) alongside a common reference (e.g., an adult’s palm), converting abstract weight units into intuitive visual cues. Portion sizes were thus reported using these culturally relevant and visually anchored units. Based on the reported intake, a Healthy Eating Adherence Score (range 0–50) was calculated by comparing each food category’s estimated weekly intake against the recommended ranges in the Dietary Guidelines for Chinese Residents (2022).

**Table A3:** Food Categories Assessed in the FFQ-25.

Food Category (English Translation)	Food Category (Original Chinese)
1. Cooked Rice	1. 米饭
2. Porridge/Congee	2. 粥、稀饭或泡饭
3. Flour-based Foods (e.g., steamed buns, bread, noodles)	3. 面粉类食物（馒头、面包、面条、饼）
4. Sweets/Pastries/Cakes	4. 甜食、点心、蛋糕
5. Deep-fried Foods (e.g., fried dough sticks)	5. 油炸食物（油条、油饼）
6. Stuffed Foods (e.g., dumplings, buns)	6. 有馅类食物（包子、馄饨、饺子）
7. Coarse Grains (e.g., millet, corn, oats, red beans)	7. 粗杂粮（包括糙米、小米、玉米、薏米、燕麦、红小豆、绿豆等）
8. Tubers (e.g., sweet potato, potato, taro)	8. 薯类（包括红薯、土豆、芋头、山药、魔芋等）
9. Dairy (milk, yogurt, milk powder)	9. 牛奶、酸奶或奶粉
10. Eggs (chicken or duck)	10. 鸡蛋或鸭蛋
11. Red Meat Dishes (pork, beef, lamb)	11. 红肉类菜肴（如猪、牛、羊肉）
12. Poultry Dishes (chicken, duck, goose)	12. 家禽类菜肴（如鸡、鸭、鹅肉）
13. Processed Meat Products (e.g., sausage, ham)	13. 加工肉制品（如香肠、熏肉、午餐肉、火腿等）
14. Soy Products (e.g., tofu, dried tofu)	14. 豆制品类菜肴（如豆腐、香干、素鸡、百叶结等）
15. Freshwater Fish and Shrimp	15. 河鲜类菜肴（如青鱼、鲈鱼、鲢鱼、河虾等）
16. Seafood	16. 海鲜类菜肴（如带鱼、鲳鱼、黄鱼、海虾等）
17. Nuts (e.g., watermelon seeds, sunflower seeds, walnuts)	17. 坚果类食物（西瓜子、葵花子、核桃、花生、开心果）
18. Dark-colored Vegetables (e.g., leafy greens, tomatoes, carrots)	18. 深色蔬菜类菜肴（如青菜、菠菜、空心菜、番茄、青椒、胡萝卜等）
19. Light-colored Vegetables (e.g., cabbage, radish, cucumber)	19. 浅色蔬菜类菜肴（如白菜、萝卜、黄瓜等）
20. Mushrooms (e.g., shiitake, straw mushrooms)	20. 菌菇类蔬菜（如蘑菇、香菇、草菇、平菇等）
21. Fruits (e.g., apple, pear, banana, orange, watermelon)	21. 水果（如苹果、梨、桃、香蕉、葡萄、橙子、橘子、西瓜等）
22. Sugar-sweetened Beverages (e.g., cola, iced tea)	22. 甜饮料（如可乐、雪碧、冰红茶、运动饮料等）
23. Beer	23. 啤酒
24. Rice Wine	24. 黄酒
25. Spirits	25. 白酒

Note: Each category followed the same format: a frequency question (“In the past month, how often did you consume [food category]?”) followed by a portion size question (“What was your typical portion size per occasion?”). Frequency options were standardized across categories. Portion size options were tailored to each food type, using units such as liang (两, ≈50 g) for most solids, milliliters (mL) for liquids, specific counts (e.g., number of eggs, bottles), or descriptive categories (e.g., “half a bottle or less”). If “Never” was selected for frequency, the portion size question was skipped automatically.

### Appendix A.5. Social–Cognitive Variables

**Table A4:** Items Measuring Social–Cognitive Variables (Intentions, Planning, and Self-Efficacy) for Physical Activity and Healthy Eating.

Construct	Chinese Item	English Item
Intention for PA	1. 我打算经常剧烈运动，使自己出汗，呼吸急促	1. I intend to vigorously exercise regularly, so that I sweat and become short of breath.
	2. 我打算定期适度运动，这样我在闲暇时就会出汗	2. I intend to be regularly and moderately active, so that I sweat a bit in leisure time.
	3. 我打算在日常生活中积极活动（散步、骑自行车、做家务和园艺）	3. I intend to be active in daily life (walking, biking, house and garden work).
Intention for HE	4. 我打算多吃些水果或蔬菜	4. I intend to eat more fruit or vegetables.
	5. 我打算饮食更均衡一些	5. I intend to eat a more balanced diet.
	6. 我打算坚持健康的饮食	6. I intend to adhere to the healthy Mediterranean diet.
Plan for PA	7. 我已经制定了一个关于什么时候、在哪里、怎样锻炼的详细计划	7. I have made a detailed plan for when, where, or how to exercise.
	8. 我已经制定了一个关于多长时间锻炼一次、和谁一起锻炼的详细的计划	8. I have made a detailed plan for how often and with whom to exercise.
Plan for HE	9. 我已经做了一个关于什么时候、在哪里、如何吃水果或蔬菜的详细的计划	9. I have made a detailed plan for when, where, or how to eat fruit or vegetables.
	10. 我已经制定了一个如何保持全面均衡的饮食的详细的计划	10. I have made a detailed plan for how to maintain an overall balanced diet.
Self-efficacy for PA	11. 我相信我可以更规律地运动，即使我必须强迫自己立即开始	11. I am sure that I can do more regular physical activity, even if I have to force myself to start immediately.
	12. 我相信我可以做更规律地运动，即使我感到强烈的不运动的诱惑	12. I am sure that I can do more regular physical activity, even if I feel a strong temptation not to exercise.
Self-efficacy for HE	13. 我相信我可以改善自己的日常营养状况，即使我必须强迫自己立即开始	13. I am sure that I can improve my daily nutrition, even if I have to force myself to start immediately.
	14. 我相信我可以改善自己的日常营养状况，即使我感受到零食和放纵的强烈诱惑	14. I am sure that I can improve my daily nutrition, even if I feel a strong temptation to snack and indulge.

Note: The relatively low Cronbach’s alpha for physical activity intention (α = 0.41) is likely due to the scale capturing intentions for behaviorally distinct activities (vigorous exercise, moderate exercise, and light-intensity daily activities). As this approach measures the broader theoretical construct of physical activity intention—encompassing multiple domains as is common in health behavior research (e.g., Fleig et al., 2015b; Schwarzer, 2008; Sniehotta et al., 2005)—the composite score was retained for analysis.
